# Comparative evaluation of sedative and anti-nociceptive effects of epidural romifidine, romifidine–lidocaine, and lidocaine in donkeys (*Equus asinus*)

**DOI:** 10.3389/fvets.2022.966715

**Published:** 2022-12-01

**Authors:** Mohamed Marzok, Adel I. Almubarak, Hussein Babiker, Mahmoud Kandeel, Sayed Fathi El-Hawari, Sabry El-khodery

**Affiliations:** ^1^Department of Clinical Scienses, College of Veterinary Medicine, King Faisal University, Al-Ahsa, Saudi Arabia; ^2^Department of Surgery, Faculty of Veterinary Medicine, Kafrelsheikh University, Kafrelsheikh, Egypt; ^3^Department of Biomedical Sciences, College of Veterinary Medicine, King Faisal University, Al-Ahsa, Saudi Arabia; ^4^Department of Pharmacology, Faculty of Veterinary Medicine, Kafrelsheikh University, Kafrelsheikh, Egypt; ^5^Department of Surgery, Faculty of Veterinary Medicine, Sohag University, Kafrelsheikh, Egypt; ^6^Department of Internal Medicine, Infectious Diseases and Fish Diseases, Faculty of Veterinary Medicine, Mansoura University, Manosura, Egypt

**Keywords:** anti-nociception, donkey, epidural, romifidine, sedation

## Abstract

**Background:**

Local and regional anesthetic procedures are valuable tools in veterinary practice. Caudal epidural administration of local anesthetic agents is widely reported for surgical interventions of the tail, anus, rectum, vulva, vagina, urethra, and bladder in the standing horse. Epidural analgesia is also obtained using various drugs such as alpha-2 adrenoceptor agonists, dissociative anesthetics, and opioids. The present study evaluates the anti-nociceptive and sedative effects of epidural administration of romifidine, a romifidine–lidocaine combination, and lidocaine alone in donkeys.

**Materials and methods:**

In a randomized prospective study, twenty-four healthy adult donkeys were assigned to four groups (three experimental and one control; *n* = 6) received either 50 μg/kg of romifidine, 0.30 mg/kg of lidocaine, combined romifidine (50 μg/kg) and lidocaine (0.30 mg/kg) diluted in 0.9% sterile normal saline solution to a total injection volume of 12 ml, or an equivalent volume of sterile saline epidurally. After epidural injection of each treatment, the onset, degree, and duration of sedation and anatomical extension of anti-nociception were documented. Observations began immediately (time 0) pre-administration and at 5, 15, 30, 45, 60, and 30-min intervals subsequently until 210 min after drug injection. Time to onset of perineal analgesia was documented every minute after the epidural injection by evaluating the animal's response to pinpricks.

**Results:**

Only romifidine and romifidine-lidocaine induced mild to moderate sedation. Romifidine, romifidine-lidocaine, and lidocaine induced complete bilateral caudal epidural analgesia with loss of sensation in the perineum, tail, inguinal region, caudal aspect of the upper hind limb, chest areas, and extended distally to the dorsal metatarsal area. Sedation lasted longer (*p* < 0.05) with romifidine (160 ± 15.4 min) than with romifidine-lidocaine (141.6 ± 13.2 min). Longer-lasting analgesia (*p* < 0.05) was obtained with romifidine (158.3 ± 9.8 min) and romifidine-lidocaine (165 ± 9.4 min) than with lidocaine (75.8 ± 8 min).

**Conclusions:**

Epidural administration of a single dose of romifidine or a combination of romifidine-lidocaine produced mild to moderate sedation and complete anti-nociception in the perineal and inguinal regions of donkeys. The clinical usefulness of epidural romifidine or romifidine-lidocaine combinations to perform obstetric procedures in donkeys needs to be assessed.

## Introduction

Local and regional anesthetic procedures are valuable tools in veterinary practice. The epidural injection of local anesthetic agents in equine clinical practice was first attempted in Germany more than a century ago to avoid the costs and risks associated with general anesthesia and recumbency (1). Currently, caudal epidural administration of local anesthetic agents is widely reported for surgical interventions of the tail, anus, rectum, vulva, vagina, urethra, and bladder in the standing horse (2). The technique is a convenient way to provide analgesia because it is simple, cost-effective, and does not require special or advanced instruments. Furthermore, the caudal epidural anesthesia technique can provide peri-operative analgesia or alleviate inflammatory, traumatic, and chronic pain of the hind limb or pelvis of the equine (3).

Epidural analgesia is also obtained using various drugs such as alpha-2 adrenoceptor agonists (2–9), dissociative anesthetics (10), and opioids (11–14) that unselectively block sensory fibers, resulting in considerable analgesia with a reduced risk of pelvic limb disorders (12, 15, 16). These drugs are administered alone or in various combinations (8).

Romifidine is the alpha-2 adrenoceptor agonist most routinely used in equine practice in certain European and Middle East countries and is used widely as a sedative and analgesic for standing surgeries, as a pre-anesthetic medication in various anesthetic protocols, as a neuraxial (epidurals or spinals) for conduction analgesia, and as a continuous rate infusion (17, 18).

In the veterinary literature, assessment of the anti-nociceptive and sedative effects of epidural romifidine alone or in combination with other drugs has been reported in horses and food animals (6, 19–22). Although a few reports have studied the outcomes of epidural dexmedetomidine and xylazine in donkeys (23, 24) to the authors' knowledge, the use of romifidine epidural analgesia in this species has not been explored. As a result, the goal of this investigation was to evaluate and compare the anti-nociceptive and sedative effects of romifidine, lidocaine, and a mixture of romifidine-lidocaine when injected into the donkey's extradural space. It was hypothesized that (1) romifidine and the combination of romifidine and lidocaine will provide effective and potent analgesia in the perineal region and (2) the duration of analgesia would be longer with romifidine- lidocaine combination than with romifidine or lidocaine alone.

## Materials and methods

### Animals

Twenty-four healthy adult donkeys were selected (right non-pregnant females, eight geldings, and eight intact males). Their age was ~3 to 7 years, and their body weights were 180 to 220 kg. All donkeys were concluded to be healthy based on physical examination and the analysis of biochemical and hematological parameters. Based on historical data, the inclusion criteria were donkeys that had not received previous extradural administrations, regional blocks, or local analgesia in the perineal region. The exclusion criteria were donkeys with unhealthy condition, unpalpable intercococcygeal space and those previously received epidural injections or local blocks in the perineal region. Each animal was identified by a microchip inserted subcutaneously into the donkey's neck *via* a hypodermic syringe. All donkeys were housed in straw-bedded horse boxes (two animals from the same sex per box) and received the same nutrition and other management practices. They had unlimited access to food and water until the start of each treatment.

### Study protocol

The Animal Care Committee of King Faisal University (approval no. KFUREC-2022-ETHICS 12) reviewed and approved this protocol in correspondence with Saudi Arabian ethical codes for studies on experimental animals. In the present study a randomized prospective study design was used. Donkeys were assigned randomly to four treatment groups, with six donkeys in each group. Randomization was carried out using the Simple randomization technique, which involved selecting random numbers. Donkeys were assigned randomly to four treatment groups, with six donkeys in each group (two non-pregnant females, two geldings, and two intact males). All investigations were conducted in a quiet room indoors (≈25°C) with natural sunlight. Two donkeys were brought and constrained in small stocks (2 m from one another) with their heads allowed free movement. Donkeys were acclimated to their surroundings in the room for 15 mins before evaluation.

The hair over the second intercoccygeal intervertebral space was clipped and shaved, and the area was cleaned with povidone–iodine. At the beginning of each experiment, the donkeys were weighed and rectal temperature (RT), heart rate (HR), and respiratory rate (RR) for each donkey were measured.

Each group received one of four treatments (all equal volumes). Treatments were either 50 μg/kg of romifidine (10 mg/mL, Boehringer Ingelheim, Vetmedica, Ingleheim, Germany), 0.30 mg/kg of lidocaine (20 mg/mL, preservative-free and vasoconstrictor-free, Pharmaceutical Solutions Industry, Jeddah, KSA), combined romifidine-lidocaine (50 μg and 0.30 mg/kg) diluted in 0.9% sterile normal saline solution (Pharmaceutical Solution Industries, Al -Khobar, KSA) to a total injection volume of 12 ml, or an equivalent volume of sterile saline.

All treatments directly administered into the extradural space between the second and third coccygeal vertebrae (the second intercoccygeal space) over approximately 20 s, using an 18-gauge, 5-cm hypodermic needle. The specific space was identified by moving the tail up and down while palpating the depression between the second and third coccygeal vertebrae. The needle was inserted into the skin surface at a 30° angle with the median plane. The detection of negative pressure with the hanging drop technique and the lack of resistance to injection were used to confirm the correct needle placement. All treatments were prepared by one person (MK) and administered by the same investigators (MM and AA), who were blinded to the drug used.

### Evaluation of epidural effects of romifidine, romifidine- lidocaine combination, and lidocaine

The onset time, duration, anatomical extension of anti-nociception, and sedation were documented after the epidural injection of each drug. Clinical observations included HR, RR, RT, and scores for sedation, anti-nociception, and ataxia, tail tone (flaccidity), anal and rectal relaxation, inspiratory sounds, penile prolapse in males, and frequency of micturition. Observations began immediately (time 0) pre-administration and at 5, 15, 30, 45, 60, and 30-min intervals subsequently until 210 min after drug injection. Tail tone (flaccidity) was detected by manual palpation of the tail, and visual assessment was used to evaluate rectal relaxation. The time interval between extradural administration and the beginning of loss of the tail tone and penile prolapse was used to determine the time of onset of tail and penile relaxation. HR was assessed by auscultation as beats per minute, RR was evaluated as the number of rising and falling movements of the ribs or flank area per minute, and RT was measured with a lubricated rectal veterinary digital thermometer.

### Assessment of anti-nociception

Anti-nociception was tested at different points, including the tail root, anus, vulva, perineum, the skin of the posterior aspect of the upper hind limb region, flank, lateral abdominal wall, chest areas, shoulder, neck, and the dorsal metatarsal area *via* pinprick test (using a 20-gauge, 2.5-cm-long hypodermic needle). This test entailed the introduction of the needle “pinpricking” into the underlying tissues (subcutaneous tissues or deep muscles) at the above-mentioned points. The needle was introduced bilaterally at a slightly different location for each time point. The skin prick wounds were swabbed and sprayed with povidone–iodine solution.

The intensity of anti-nociception was judged using a whole-number scoring system from 0 to 3 as described in donkeys in a previous study (25): 0 = no analgesia (forceful reaction to painful stimulation, such as the vigorous motion of the animals' limb); 1 = mild analgesia (moderate reaction, such as moving the head toward the site of stimulation); 2 = moderate analgesia (very weak and intermittent response); and 3 = complete analgesia (no response to painful stimulation). The time interval between extradural administration and loss of sensation in the perineum (score= 1) was used to determine the time of onset of anti-nociception. Time to onset of perineal analgesia was documented every minute after the epidural injection by evaluating the animal's response to pinpricks. The time interval between the loss (score ≥1) and reoccurrence of reaction to nociceptive stimuli (score = 0) was used to determine the duration of the anti-nociceptive effect.

Only in the perineum, nociceptive responses (NRs) were also assessed *via* an electric impulse-based stimulus delivered by a remotely activated neuromuscular electrical stimulator (FS-204-EMS-2CH, Finesun, Guangdong, China) [Stimulus intensity level (1–10); output voltage from 3.2 to 148.4 V; duration 1.3 s]. The perineal region of each donkey was thoroughly rinsed with clean water and scrubbed with alcohol (70%) before the trials. Two adhesive circle-shaped electrodes (25-mm diameter) were placed approximately 4 cm apart, on the skin of the perineal area with water-soluble conductive gel. During the test, each donkey was exposed to an electrical stimulus (Constant current mode) of gradually increasing electrical intensity voltage (V) until a consistent NR was detected and the respective voltage depth level was reported (Table 1). Positive NRs were described as deliberately avoiding movements of the tail, limbs, trunk, head, and neck, attempting to kick, and turning the head toward the stimulus (pinprick or electrical) site. Skin twitching was not considered an avoidance reaction. The investigator stopped the stimulus immediately as the donkey displayed any active pain (noxious) reaction. The nociceptive threshold (NT) was defined as the lowest intensity level at which a positive NR was first observed. If the NT was significantly higher than the NT measured at time 0, a donkey was considered to have perineal analgesia at that time point. The donkeys' eyes were wrapped at the time of stimulation to prevent the donkey from seeing the operator at the moment the electrical stimulus was applied and to prevent any visibly generated response.

**Table 1:** Output voltage and duration values for each intensity level of electrical stimulus.

**Stimulus intensity level**	**Output voltage (Vpp)**
1	3.2
2	3.5
3	12
4	31.8
5	55.2
6	78.4
7	95.2
8	116
9	134
10	148.4

### Assessment of sedation

In each donkey, the sedative effect for each treatment was assessed using a four-point descriptive scale (25): 0 = no sedation (aware, sensitive to waving the hands near the face of the animal, behaving normally); 1 = mild sedation (minimal lowering of head and lips, reduced alertness with slightly decreased reaction to waving hands near the face of the animal); 2 moderate sedation (sluggishness, occasional response to waving the hands near the face of the animal, moderate lowering of head and lips, palpebral ptosis and deviation of the neck, ears pointing out and lower ear carriage); and 3 = deep sedation (marked sluggishness, loss of response to waving the hands near the head of the animal, an obvious drop of head and lips, marked palpebral ptosis and deviation of the neck and pronounced ears tips separation and lower ear carriage). The interval time from the extradural administration to the onset of sedation (score ≥1) was considered the sedation onset time. The time from the onset of sedation to the return of the sedation score to zero was estimated as the duration of sedation (in minutes).

### Assessment of motor effects (ataxia)

The degree of ataxia in all donkeys was assessed by walking them out of the stocks and observing the position of their hind limbs, how much they swayed, and the extent of fetlock rolling or knuckling over. Ataxia was scored on a 4-point scale (25): 0 = normal, 1 = slight or mild (slight or intermittent wide stance of hind legs, slight swaying or stumbling, but capable of wandering), 2 = moderate (pronounced swaying, frequent wide stance of hind legs, frequent fetlock knuckling, walking with extreme incoordination), or 3 = severe (constant fetlock knuckling, recumbency or falling while walking). The time elapsed from the extradural administration to the onset of ataxia (score ≥1) was considered the time of onset of ataxia. The time from the onset of ataxia to the return of the ataxia score to zero was estimated as the duration of ataxia (minutes).

The same observer (AM) evaluated anti-nociception, ataxia, and sedation in all animals and was completely unaware of the treatments administered to each donkey or the intensity (voltage) of the electrical stimulus applied to the perineal area.

### Data analysis

Statistical analyses were performed using the SAS program version 9.2 (GMP, SAS, Inc. USA). For variables presented as scores (analgesia, sedation, and ataxia), a non-parametric Kruskal–Wallis test followed by Tukey honestly significant difference test (HSD) was used at each time point, and the results are presented as median and range. However, for variables with continuous data (HR, RR, and RT), a repeated-measures ANOVA was used to evaluate the effect of time and treatment and the interaction between time and treatment. The results are presented as mean ± SD. The onset and duration were evaluated using a one-way ANOVA with *post-hoc* Duncan multiple comparison test. For all statistical analyses, *p*-values < 0.05 were considered significant.

## Results

The epidural injection was easily and successfully performed in all donkeys without any complications noted after epidural injection. No precipitation, turbidity or change in color occurred in the romifidine-lidocaine mixture.

### Anti-nociception

**Figure 1:**
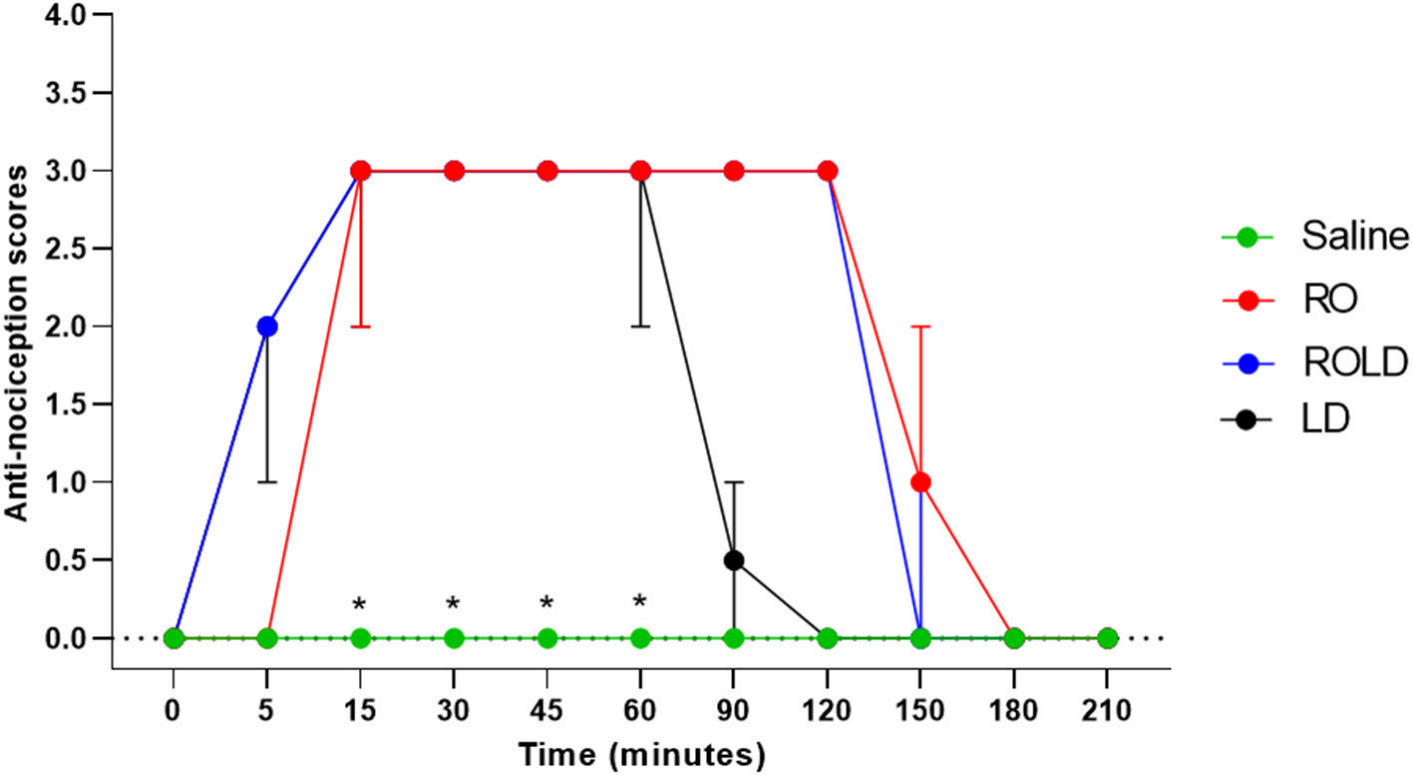
Anti-nociception scores (median and range) in the tail, perineum, inguinal region, caudal aspect of the upper hind limbs, flank and chest regions (Response to “pin-prick” stimulation) pre- epidural and post- epidural administration of saline (*n* = 6), romifidine (RO) (50 μg kg−1) (*n* = 6), romifidine-lidocaine (ROLD) (50 μg and 0.30 mg kg−1) (*n* = 6) and lidocaine (LD) (0.3 0 mg kg−1) (*n* = 6) in donkeys. *: Saline differ significantly from RO, LD, and ROLD combination.

**Figure 2:**
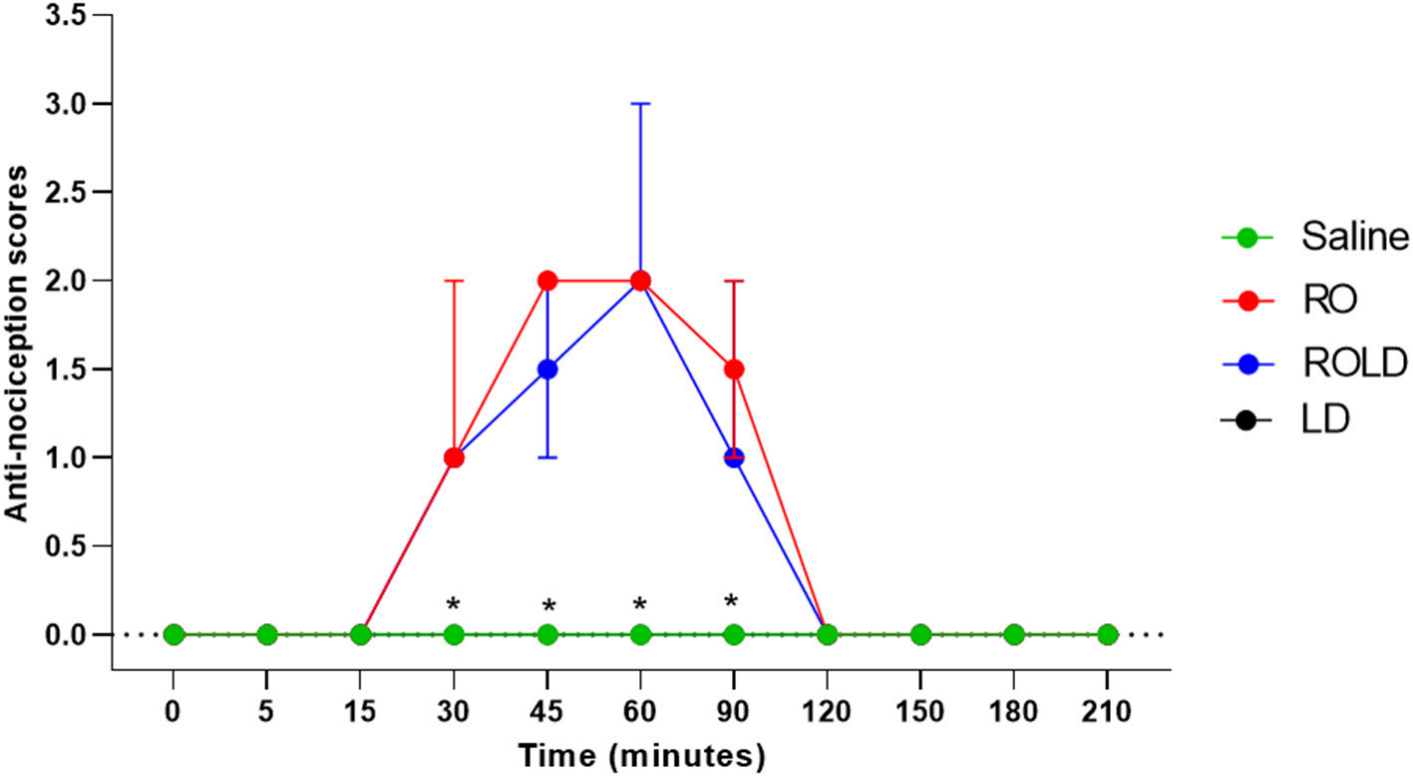
Anti-nociception scores (median and range) in the dorsal metatarsal and ventral abdominal wall regions (Response to “pin-prick” stimulation) pre- epidural and post- epidural administration of saline (*n* = 6), romifidine (RO) (50 μg kg−1) (*n* = 6), romifidine-lidocaine (ROLD) (50 μg and 0.30 mg kg−1) (*n* = 6) and lidocaine (LD) (0.3 0 mg kg−1) (*n* = 6) in donkeys. *: Saline and LD differ significantly from RO and ROLD combination.

**Figure 3:**
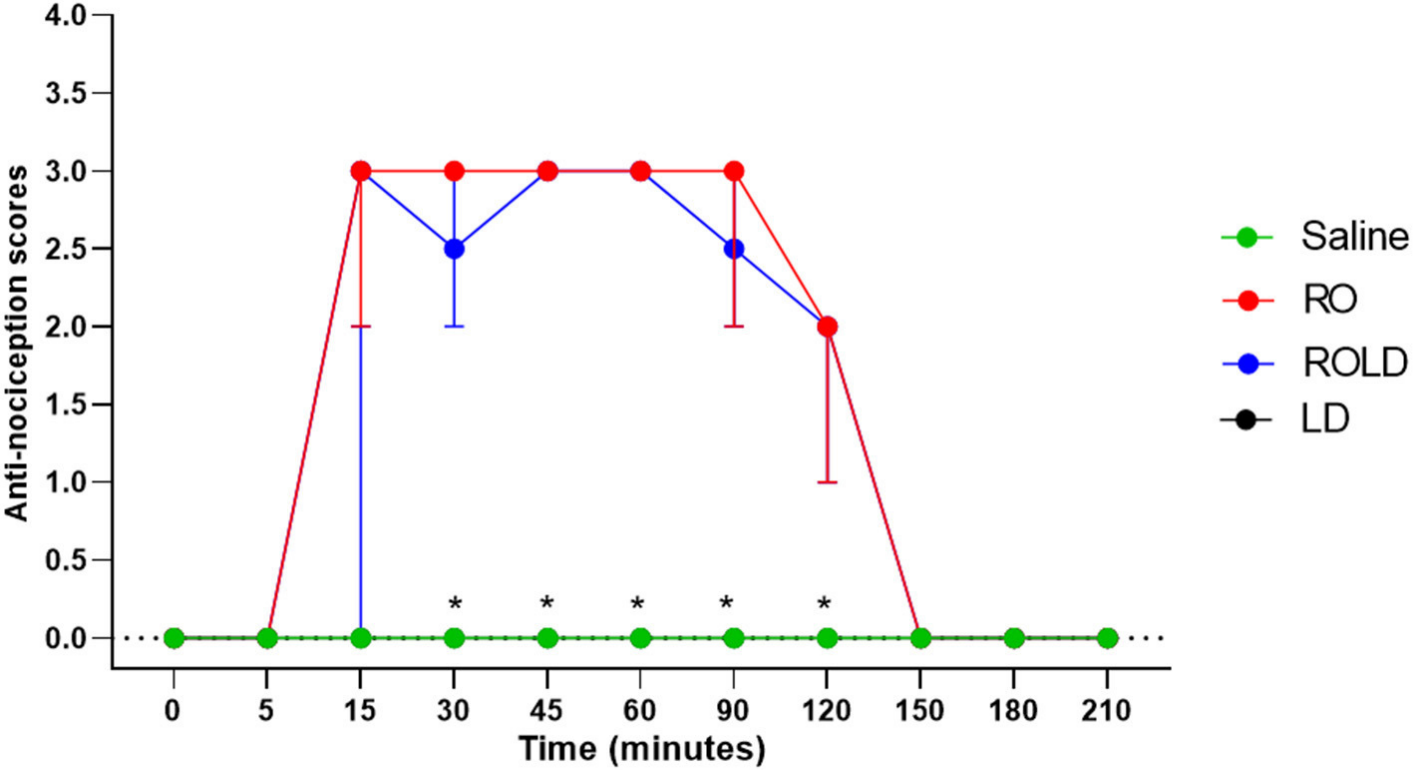
Anti-nociception scores (median and range) in the hind limb stifle and hock regions (Response to “pin-prick” stimulation) pre- epidural and post- epidural administration of saline (*n* = 6), romifidine (RO) (50 μg kg−1) (*n* = 6), romifidine-lidocaine (ROLD) (50 μg and 0.30 mg kg−1) (*n* = 6) and lidocaine (LD) (0.3 0 mg kg−1) (*n* = 6) in donkeys. *: Saline and LD differ significantly from RO and ROLD combination.

Based on treatment-time interaction, the degree of anti-nociception showed a significant variation between the four treatments and with the progression of time. All donkeys injected with normal saline epidurally did not show significant changes in the nociceptive reflexes. Romifidine, romifidine-lidocaine, and lidocaine treatments induced complete bilateral caudal epidural analgesia with loss of sensation in the tail, perineum, inguinal region, and the caudal aspect of the upper hind limb (Figure 1). However, the anatomic extent of anti-nociception in all donkeys that received either romifidine or romifidine-lidocaine combination was extended cranially to the chest areas and distally to the dorsal metatarsal area (Figures 2, 3). Romifidine, romifidine-lidocaine, and lidocaine treatments resulted in a maximum degree of anti-nociception (score = 3) but demonstrated different onset times, with varying lengths and locations (Figures 1–3).

Donkeys who received lidocaine experienced the shortest duration to the onset of perineal anti-nociception (3.6 ± 0.8 min), followed by those who received romifidine-lidocaine combination (4.0 ± 0.9 min) and romifidine (7.6 ± 0.8 min). Romifidine and romifidine-lidocaine combination resulted in a significantly (*p* < 0.05) longer period of analgesia (158.3 ± 9.8 min and 165 ± 9.4 min, respectively) than lidocaine (75.8 ± 8) (Table 2). In the perineum, tail, upper pelvic limb, flank, and chest region, romifidine and romifidine-lidocaine combination treatments provoked a longer anti-nociception effect than in the dorsal metatarsal area, ventral abdominal wall, stifle, and hock regions (Figures 1–3). In contrast to saline, administration of romifidine, romifidine-lidocaine and lidocaine treatments induced a significant increase (*p* < 0.05) in the NT to electrical stimulation of the perineal region. NT increased from 15 to 120 min post administration of lidocaine. However, it increased from 30 to 180 min post administration romifidine, and romifidine-lidocaine combination treatments. In the lidocaine treatment group, the greatest NT levels appeared between 45 and 90 min, but the highest levels for romifidine and romifidine-lidocaine groups appeared between 45 and 150 min after epidural injection (Figure 4).

**Table 2:** Onset and duration of perineal anti-nociception, sedation, ataxia, tail relaxation (flaccidity) and penile prolapse (Mean ± Standard deviation) following epidural injection of RO (*n* = 6), ROLD (*n* = 6), LD (*n* = 6), and (*n* = 6) in donkeys.

**Variable**	**RO**	**ROLD**	**LD**	**Saline**
**Anti-nociception**				
Onset	7.6 ± 0.8^a^	4 ± 0.9^b^	3.6 ± 0.8^b^	^#^
Duration	158.3 ± 9.8^a^	165 ± 9.4^a^	75.8 ± 8^b^	^#^
**Sedation**				
Onset	6.3 ± 1.2	6.5 ± 1.0	^#^	^#^
Duration	160 ±15.4^a^	141.6 ±13.2^b^	^#^	^#^
**Ataxia**				
Onset	12.8 ± 1.9	14 ± 1.2	^#^	^#^
Duration	149.5 ± 12	144.1 ± 15.3	^#^	^#^
**Penile relaxtion (prolapse)**				
Onset	21 ± 4	19 ± 2.5	^#^	^#^
Duration	80.5 ± 5.9	79.5 ±3.9	^#^	^#^
**Tail relaxation (tone)**				
Onset	4 ± 0.8	4 ± 1	3.8 ± 0.9	^#^
Duration	110.5 ± 3.3^a^	109.5 ± 3.9^a^	70 ± 2.7^b^	^#^

a, b c: Means with different superscript letters at the same raw are significantly different at p < 0.05.

# indicates that treatment provided no effect.

**Figure 4:**
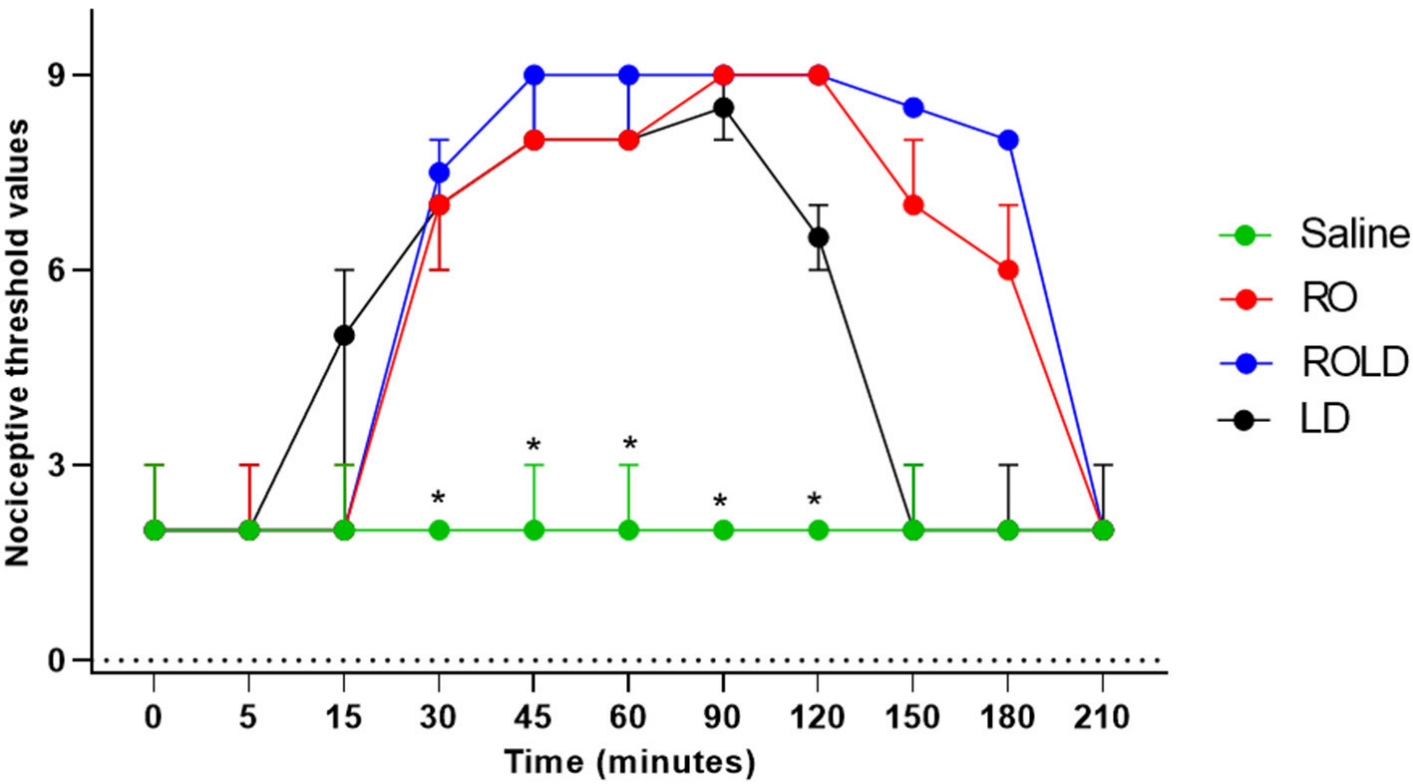
Nociceptive threshold values (median and range) in the perineum pre- epidural and post- epidural administration of saline (*n* = 6), romifidine (RO) (50 μg kg−1) (*n* = 6), romifidine-lidocaine (ROLD) (50 μg and 0.30 mg kg−1) (*n* = 6) and lidocaine (LD) (0.3 0 m kg−1) (*n* = 6) in donkeys. *: Saline differ significantly from RO, LD, and ROLD combination.

### Sedation

**Figure 5:**
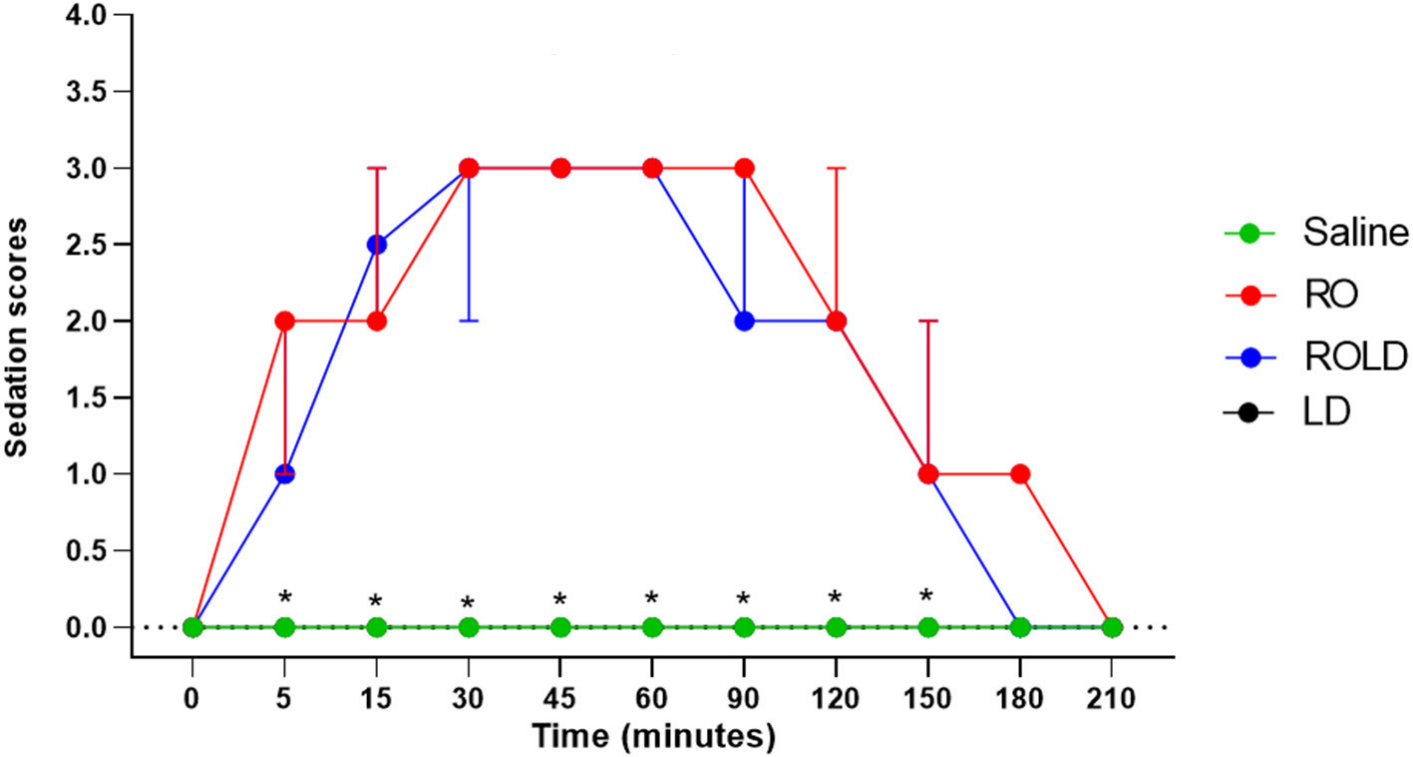
Sedation scores (median and range) pre- epidural and post- epidural injection of saline (*n* = 6), romifidine (RO) (50 μg kg−1) (*n* = 6), romifidine-lidocaine (ROLD) (50 μg and 0.30 mg kg−1) (*n* = 6) and lidocaine (LD) (0.3 0 mg kg−1) (*n* = 6) in donkeys. *: Saline and LD differ significantly from RO and ROLD combination.

Donkeys in the romifidine and romifidine-lidocaine groups showed significant changes in sedation scores (*p* < 0.05) compared to the kidocaine and saline groups. Lidocaine and saline elicited no sedative effect. Both romifidine and romifidine-lidocaine combination induced mild to moderate sedation (score = 1–2) within 5 min after epidural administration (Figure 5). The romifidine duration of sedation was significantly (*p* < 0.05) longer than romifidine-lidocaine combination (160 ± 15.4 min and 141.6 ± 13.2 min, respectively) (Table 2).

The maximum sedation detected in this study was a score of 3 recorded between 30 and 90 min after epidural injection of both romifidine and romifidine-lidocaine combination. By 180 min after epidural administration, all donkeys behaved normally, no longer sedated and aware of their surroundings.

### Ataxia/motor incoordination

**Figure 6:**
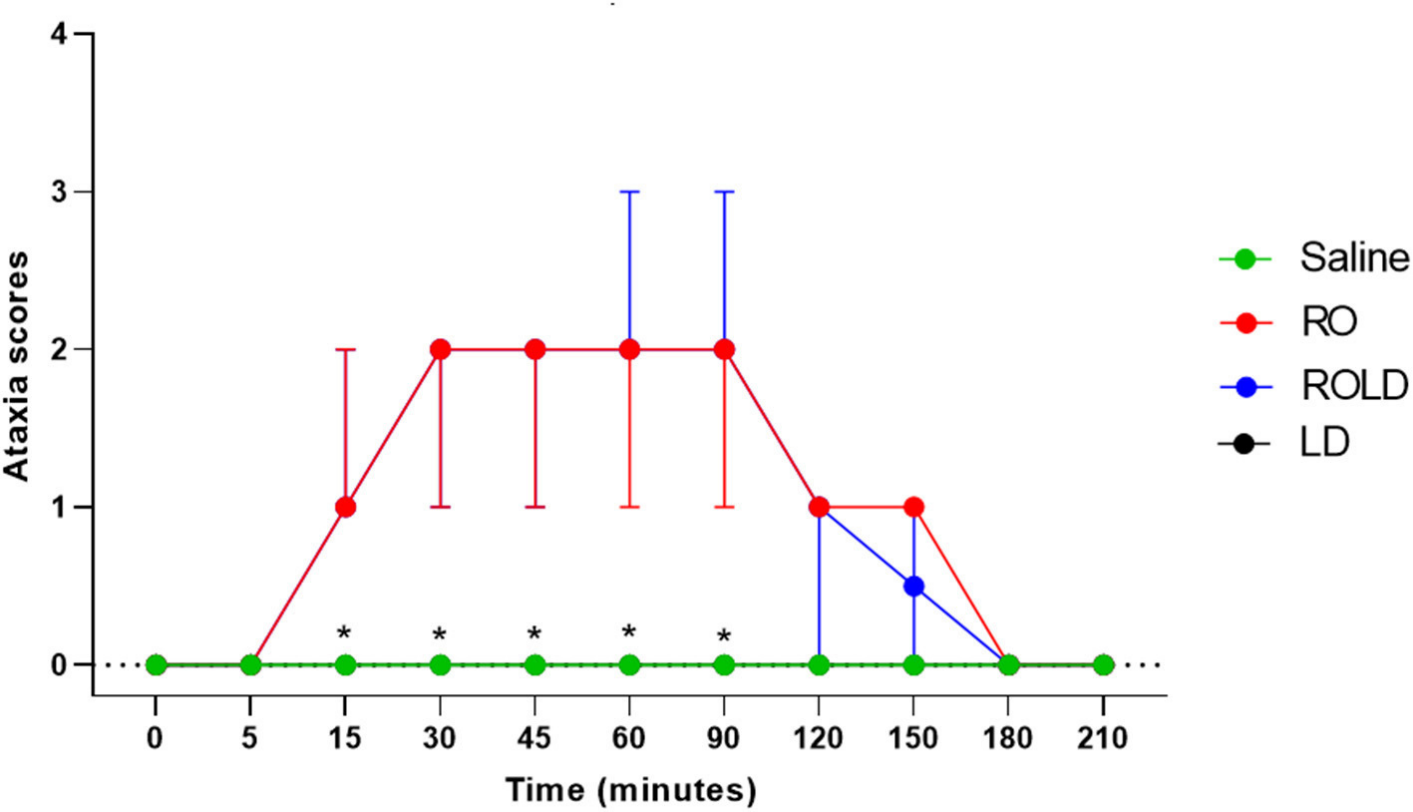
Ataxia scores (median and range) pre- epidural and post- epidural injection of saline (*n* = 6), romifidine (RO) (50 μg kg−1) (*n* = 6), romifidine-lidocaine (ROLD) (50 μg and 0.30 mg kg−1) (*n* = 6) and lidocaine (LD) (0.3 0 mg kg−1) (*n* = 6) in donkeys. *: Saline and LD differ significantly from RO and ROLD combination.

Donkeys in the treatment romifidine and romifidine-lidocaine groups showed significantly different ataxia scores than those in the treatment saline and lidocaine groups (*p* < 0.05). Mild (score = 1) to moderate (score = 2) ataxia was noted in romifidine and romifidine-lidocaine treated donkeys 15 min post administration and lasted until 90 min, but was not noted in the donkeys treated with saline or lidocaine epidural administration (Figure 6). Two donkeys of romifidine-lidocaine group suffered from severe ataxia (score = 3).

### Clinical evaluation

**Figure 7:**
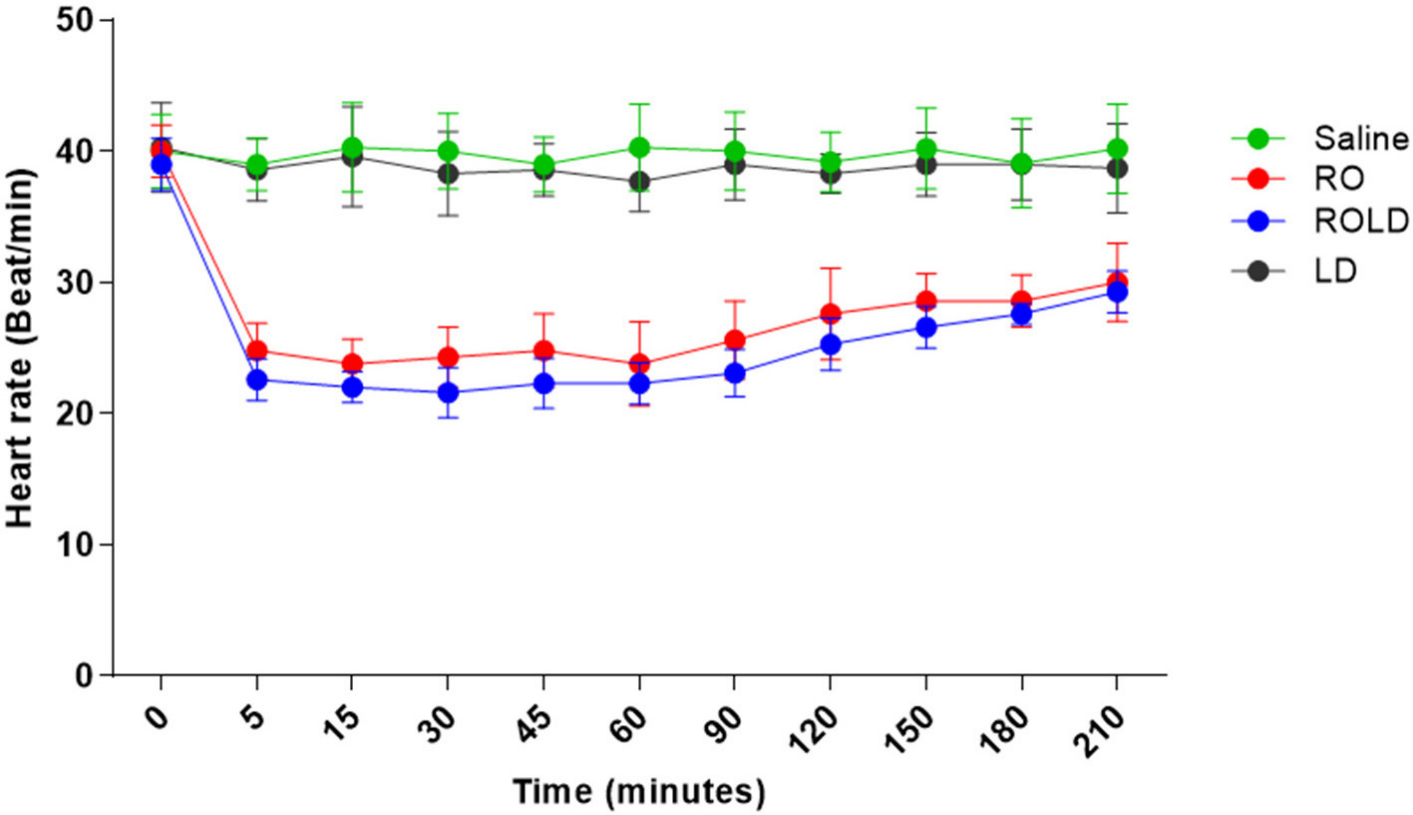
Heart rate (Beat / Min.; mean and standard deviation) pre- epidural and post- epidural administration of saline (*n* = 6), romifidine (RO) (50 μg kg−1) (*n* = 6), romifidine-lidocaine (ROLD) (50 μg and 0.30 mg kg−1) (*n* = 6) and lidocaine (LD) (0.3 0 mg kg−1) (*n* = 6) in donkeys. Wilks Lambda test for time x treatment, *P* < 0.01, indicates significant changes in respiratory rate under the effect of treatment and time. However, sphericity assumed test indicates the effect of time (within-treatment). Wilks Lambda test for time x treatment interaction *P* < 0.01. Sphericity assumed test for the effect of time (within-treatment), *P* < 0.05.

**Figure 8:**
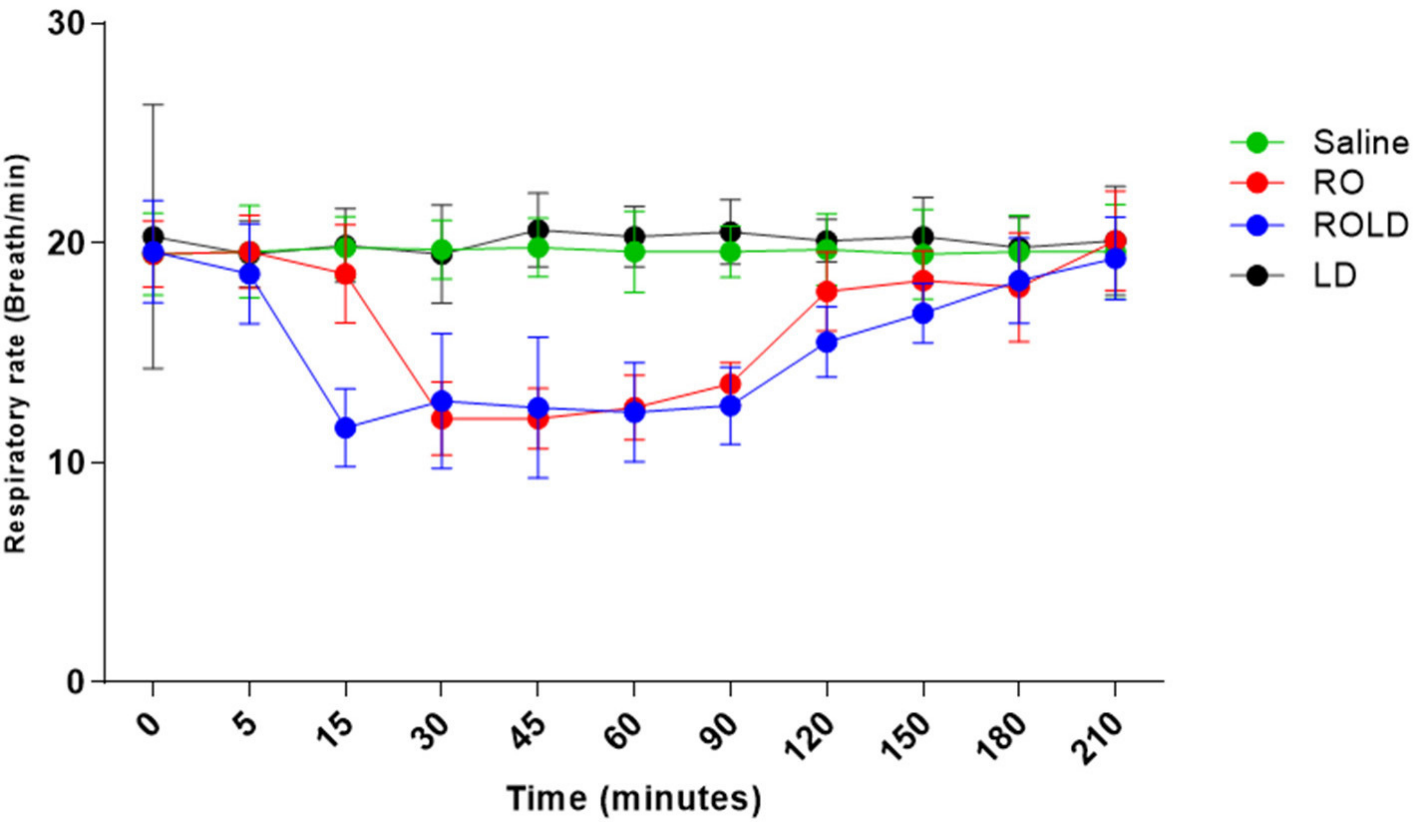
Respiratory rate (respiratory cycle / Min mean and standard deviation) pre-epidural and post-epidural administration of saline (*n* = 6), romifidine (RO) (50 μg kg−1) (*n* = 6), romifidine-lidocaine (ROLD) (50 μg and 0.30 mg kg−1) (*n* = 6) and lidocaine (LD) (0.3 0 mg kg−1) (*n* = 6) in donkeys. Wilks Lambda test for time x treatment, *P* < 0.01, indicates significant changes in respiratory rate under the effect of treatment and time. However, sphericity assumed test indicates the effect of time (within-treatment). Wilks Lambda test for time x treatment interaction *P* < 0.01. Sphericity assumed test for the effect of time (within-treatment), *P* < 0.01.

Heart rate and respiratory rate differed significantly after epidural injection of romifidine and romifidine-lidocaine combination (Wilks' lambda for treatment-time interaction, *p* < 0.05) compared to the baseline value (Figures 7, 8). The lowest HR occurred 45–60 min post-injection in both romifidine and romifidine-lidocaine groups. However, donkeys who had received lidocaine or saline did not show significant changes in both RR and HR rates. The HR and RR did not change significantly between romifidine and romifidine-lidocaine treatments or between study times within the same treatment. The RT remained constant compared to the baseline value at all times, and no significant differences were found between the four treatments.

Tail relaxation (flaccidity), anal and rectal relaxation were observed in all donkeys at 2–5 min and remained for 70, 110, and 109 min post administration of lidocaine, romifidine, and romifidine-lidocaine combination, respectively (Table 2). Penile relaxation (prolapse) was also noted in all the male donkeys at 19–21 min and remained until 79.5–80.5 min post administration of both romifidine and romifidine-lidocaine combination treatments (Table 2). Frequent urination was also observed in both of these treatment groups between 80 and 120 min, and all donkeys urinated more than once (range 3–5 times).

No treated donkey showed any clinical evidence of discomfort. Moreover, some side effects were reported post-administration for all treatments, frequent snorting or sneezing (range 5–6 times) was observed between 15 and 60 min post-administration of both romifidine and romifidine-lidocaine combination.

## Discussion

The objective of this randomized prospective study was to evaluate the sedative and anti-nociceptive effects of epidural romifidine, lidocaine, and romifidine-lidocaine combination in donkeys. Our findings indicate that epidural injections of romifidine and romifidine-combination had significant sedative, anti-nociceptive and clinicophysiological effects on donkeys. There have been no reports of the usage of epidural romifidine in donkeys. While, few studies found that IV administration of romifidine at different doses provides comparable levels of both sedation and anti-nociception, but only anti-nociception was dosage dependent (26–29).

In veterinary anesthesia, multimodal anti-nociception therapies in which an alpha-2 adrenoceptor agonist is combined with a local analgesic (such as lidocaine) have been shown to produce an effective analgesic interaction with minimum side effects (5, 30). The dosage of romifidine used in this study was derived primarily from previous research conducted in cattle, goats, and horses (6, 22, 31) and unpublished pilot studies.

In the present study, epidural romifidine and romifidine-lidocaine combination were expected to produce analgesia similar to that produced in horses (6). Epidural administration of romifidine alone in one previous study provided no analgesia in 5 of 8 horses studied and inadequate analgesia for surgical procedures in the remaining three horses (32). However, inadequate or moderate perineal analgesia has been noted post epidural administration of a combination of morphine and romifidine (6). The results of this study indicate that epidural administration of romifidine and romifidine-lidocaine combination can induce maximum perineal anti-nociception (score = 3) in all donkeys. Furthermore, the epidural injection of romifidine and romifidine-lidocaine combination offers complete anti-nociception of the tail, perineum, inguinal area, caudal aspect of the upper hind limb, chest areas, flank, and dorsal metatarsal area. These results are similar to those previously reported in large ruminants (31, 33). Similarly, previous studies reported a significant degree of perineal analgesia after epidural injection of xylazine and dexmedetomidine in donkeys but analgesia was only assessed in the perineal region (24).

Only a few recent studies have been published regarding the use of alpha-2 adrenoceptor agonists for epidural application in donkeys. Xylazine and dexmedetomidine have been shown to provide effective anti-nociceptive effects with no or minimal adverse effects (24). Epidural administration of alpha-2 adrenoceptor agents resulted in analgesia due to stimulation of both presynaptic and postsynaptic alpha-2 adrenergic receptors in the spinal dorsal horn. This stimulation causes suppression of the central nervous system's transmission of afferent nociceptive impulses and a decrease in interneuron transmission of norepinephrine and substance-p, resulting in reduced neural activities and anti-nociception (20, 33, 34).

The intensity of anti-nociception elicited by romifidine in the present study could also be attributed to systemic action after absorption through vascular or lymphatic structures in the epidural space. Intravenous romifidine has been shown to produce similar levels of anti-nociception in donkeys (26–29). Romifidine acts on the alpha-2 adrenergic receptors in the brain and spinal cord to produce its anti-nociceptive effect (35). In the study reported here, romifidine was expanded in a relatively large volume of normal saline, with the expectation that the drug would migrate cranially to the sacral and caudal lumbar spinal cord segments, implying that their actions was local effect.

In the current study, the onset of anti-nociception after epidural administration of romifidine occurred in 7.66 ± 0.81 min. These findings were similar to a previous study that reported that epidural administration of dexmedetomidine in donkeys induced a significantly rapid onset of action (5.8 ± 2.04 min) (24). Meanwhile, this effect was significantly shorter than that noted post epidural injection of different doses of xylazine (0.17, 0.35, 0.2 mg/kg) in donkeys (24.20 ± 3.56 min, 11.9 ± 2.1 min, 14.2 ± 2.1 min) (23, 24, 36).

In terms of the duration of anti-nociception, the prolonged duration of anti-nociception reported in this study after epidural romifidine and romifidine-lidocaine combination than lidocaine alone is consistent with previously observed results of other alpha-2 adrenoceptor agonists in donkeys (24). The decreased duration of anti-nociception produced by epidurally injected lidocaine may be attributed to vasodilation and greater absorption of the medication from the spinal cord into the systemic blood circulation caused by lidocaine's sympathetic blockade (10).

In both romifidine and romifidine-lidocaine groups, anti-nociception was first observed in the tail and perineum. It progressed cranially to the flank and distally to the dorsal metatarsal area. Also, the intensity and duration of anti-nociception were greater for the tail, perineum, inguinal, caudal aspect of the upper hind limbs, flank, and chest areas than in the stifle, hock, dorsal metatarsal regions, and ventral abdominal wall. This observation is most likely related to the cranial dissemination of a large amount of the drug to the caudal lumbar and sacral spinal cord segments. The reason for the short-term duration of anti-nociception of both romifidine and romifidine-lidocaine combination treatments on the dorsal metatarsal region compared to other areas is unknown. The extreme sensitivity of the dorsal metatarsal region may justify the shorter-acting anti-nociceptive effect.

In veterinary anesthesiology, alpha-2 agonists are often used to cause sedation and are classified as sedatives and analgesics. In this study, all donkeys who received epidural romifidine or romifidine-lidocaine combination showed decreased spontaneous activity with mild to moderate degree of sedation characterized by head and lips drop, palpebral ptosis, deviation of the neck, and decreased reaction to waving the hands in ahead of the animal. Sedation and other clinical effects after epidural administration of alpha-2 adrenoceptor agonists are expected to be produced via the rapid systemic uptake from the vascular or lymphatic structures in the epidural space and/or dissemination into the cerebrospinal fluid. This uptake is followed by cranial spreading to the CNS, causing CNS depression by engaging both the central and peripheral presynaptic and postsynaptic alpha-2 adrenoceptors. The further release of noradrenalin, required for arousal, is then blocked (8, 37). Sedation may also be attributed to inhibition of motivating activity of locus coeruleus, or “blue spot” neurons, which is a small nucleus situated deep in the pons of the brainstem involved in many fundamental behavioral processes such as the sleep–wake cycle and physiological reactions to anxiety and stress (38).

No significant difference was detected in the onset of sedation between romifidine and romifidine-lidocaine groups. The rapid onset of the sedative effect of both romifidine and romifidine-lidocaine treatments groups (6.3 ± 1.2 min and 6.5 ± 1.0 min) may be attributed to romifidine's high lipid solubility (39). In contrast, the onset of sedation was significantly prolonged (15.0 ± 0.0 min and 14.20 ± 4.2 min) following epidural administration of dexmedetomidine and xylazine in donkeys (24). The duration of sedation was significantly longer with romifidine (160 ± 15.4 min) than romifidine-lidocaine combination (141.6 ± 13.2 min). These results are not consistent with those of a previous investigation (6) in which epidural administration of romifidine (30 or 60 μg/kg combined with 0.1 mg/kg morphine) in horses caused a similar degree of sedation, but the average length of the effect was shorter (75–90 min). This variation may be attributed to the species difference, and slower metabolism of romifidine in donkeys after epidural injection.

The authors' findings regarding tomifidin's sedative properties in donkeys are identical to those observed for equines after epidural injection of alpha-2 adrenoceptor agonists (4, 8, 15). Substantial head and neck muscle relaxation is followed by a drop of the head, neck, ears, and lips, resulting in greater head lowering. This muscular relaxation could be attributed to motor fiber blockage, resulting in tail flaccidity, penile protrusion, and anal sphincter relaxation (40). Previous research found that all alpha-2 adrenoceptor agonists exert similar local anesthetic effects on the spinal cord nerve roots (41), and when epidurally injected, they have a local inhibitory effect on A-fibers, thought to be responsible for motor function (42).

A significant mild to slight degree of ataxia was observed, which could be attributed to the coupled systemic effects of muscular relaxation and sedation of alpha-2 agonists (43). Alpha-2 adrenoceptor agonists, in particular, inhibit sensory nerve fibers while not affecting motor fibers (44). These results are consistent with those obtained for xylazine and dexmedetomidine in donkeys (24). The romifidine or romifidine-lidocaine combination doses selected in the present study did not cause any animals to be recumbent or fall. These findings might be inconsistent with observations in cattle and buffaloes, in which recumbency was reported after epidural injection of the same dose in some cases (33).

The RT in donkeys did not change after the administration of all treatments. Alpha-2 agonists may improve body temperature maintenance by causing superficial vascular constriction and central recirculation of blood, resulting in less cutaneous heat loss (37). In contrast, a significant decrease in the RT (*p* < 0.05) was reported in donkeys after epidural administration of xylazine (23). This observed hypothermia was attributed to xylazine providing generalized sedation, muscle relaxation, depression of the CNS's thermoregulatory centers, and decreased basal metabolic rate.

The most common adverse side effects of systemic injection of alpha-2 adrenoceptor agonists include dose-dependent respiratory and cardiovascular depression (18). Epidural injection of romifidine and romifidine-lidocaine combination treatments resulted in a significant reduction in RRs and HRs. The observed RR decrease may be related to the systemic uptake of these drugs, causing sedation and a centrally mediated depression of ventilation (or the respiratory center) or CNS depressant effects (45, 46). The decrease in heart rate after epidural administration of romifidine might be attributed to central stimulation mediated through the vagus nerve and reduction in the sympathetic tone due to the decreased presynaptic release of norepinephrine (47). Similar findings have been recorded following epidural administration of romifidine and xylazine in the horse (40, 48). In contrast, no significant changes in both RRs and HRs were observed after epidural administration of both xylazine and dexmedetomidine in donkeys (24).

Frequent micturition was noticed following romifidine and combined romifidine-lidocaine administration. The higher micturition frequencies could be due to the inhibition of antidiuretic hormone release and hyperglycemia from hypoinsulinemia (37). The frequent snoring or sneezing observed in this study post-administration of both romifidine and romifidine-lidocaine combination could be attributed to a prolonged period of lower head carriage combined with upper airway relaxation.

The main limitations of the current study include the use of only one romifidine dose. This component of the research design prevents us from evaluating romifidine's dose-dependent anti-nociceptive effects in donkeys. Furthermore, a lack of knowledge on the pharmacokinetics of romifidine in donkeys presents a challenge to identify and explain some of the drugs' clinical effects. Second, only clinically normal donkeys in an experimental model was studied, which may not respond similarly to donkeys requiring surgery or experiencing painful conditions. In addition, there is no valid standardized protocol for the quantitative measurement of nociception in donkeys. Using such a protocol would permit the anti-nociceptive effects of new analgesic drugs and analgesic techniques to be accurately assessed and improved. However, we are confident that this trial demonstrated the essential characteristics of romifidine for potential application in donkey pain management. In addition, walking of donkeys outside the stock to assess ataxia my cause possible changes in sedation scores. Moreover, the use of pinprick test and electrical stimulation in the perineal region may affect analgesia scores. When designing future studies, researchers should keep these limitations in mind.

## Conclusion

This study demonstrated that, the epidural administration of a single dose of romifidine or the combination romifidine-lidocaine produced a very effective sedative effect and a rapid onset and a long duration of complete bilateral caudal epidural analgesia with no adverse effects compared to lidocaine alone in donkeys. These findings suggest that using romifidine or romifidine-lidocaine combinations in clinical practice could result in a very effective and safe method of completing many surgical and obstetrical procedures. However, more studies are needed to examine withdrawal times, spinal toxicity, cardiopulmonary adverse effects and to assess whether the analgesia is sufficient for a particular surgical procedures or for alleviating postoperative pain before any final recommendations can be made.

## Data availability statement

The original contributions presented in the study are included in the article/supplementary material, further inquiries can be directed to the corresponding author/s.

## Ethics statement

The animal study was reviewed and approved by the animal study was reviewed and approved by the Ethics Committee of King Faisal University (Approval No. KFU-REC-2022- ETHICS12).

## Author contributions

Conceptualization: MM and AA. Methodology, investigation, writing—original draft preparation, supervision, and funding acquisition: MM. Software: MK. Validation: MM, HB, and MK. Data curation: HB. Writing—review and editing: SE-k, HB, and MK. Visualization and project administration: AA. All authors have read and agreed to the published version of the manuscript.

## Funding

This work was supported through the Annual Funding track by the Deanship of Scientific Research, Vice Presidency for Graduate Studies and Scientific Research, King Faisal University, Saudi Arabia [Project No. AN000555].

## Conflict of interest

The authors declare that the research was conducted in the absence of any commercial or financial relationships that could be construed as a potential conflict of interest.

## Publisher's note

All claims expressed in this article are solely those of the authors and do not necessarily represent those of their affiliated organizations, or those of the publisher, the editors and the reviewers. Any product that may be evaluated in this article, or claim that may be made by its manufacturer, is not guaranteed or endorsed by the publisher.

## References

[1] Hall LW. Spinal Analgesia. In:Hall LW, editor. Wright's Veterinary Anaesthesia and Analgesia. 7th ed. London: Bailliere Tindall (1971). p. 102-103

[2] Skarda R Grosenbaugh D Muir W. Caudal regional anesthesia in horses. Equine Vet Educ. (2005) 15:108–16. 10.1111/j.2042-3292.2005.tb01836.x

[3] Sysel AM Pleasant RS Jacobson JD Moll HD Modransky PD Warnick LD. Efficacy of an epidural combination of morphine and detomidine in alleviating experimentally induced hindlimb lameness in horses. Vet Surg. (1996) 25:511–8. 10.1111/j.1532-950X.1996.tb01452.x 8923731

[4] Dória RG Valadão CA Duque JC Farias A Almeida RM Netto AC. Comparative study of epidural xylazine or clonidine in horses. Vet Anaesth Analg. (2008) 35:166–72. 10.1111/j.1467-2995.2007.00357.x 18275488

[5] Grubb TL Riebold TW Huber MJ. Comparison of lidocaine, xylazine, and xylazine/lidocaine for caudal epidural analgesia in horses. J Am Vet Med Assoc. (1992) 201:1187–90. 10.1111/j.1467-2995.1991.tb00580.x 1429156

[6] Natalini CC Paes SD Polydoro ADS. Analgesic and cardiopulmonary effects of epidural romifidine and morphine combination in horses. J Equine Vet Sci. (2021) 102:103459. 10.1016/j.jevs.2021.103459 34119202

[7] Robinson EP Natalini CC. Epidural anesthesia and analgesia in horses. The veterinary clinics of North America. Equine Pract. (2002) 18:61–82. 10.1016/S0749-0739(02)00010-X 12064183

[8] Rønnow Kjærulff LN Lindegaard C A. narrative review of caudal epidural anesthesia and analgesia in horses. Part 2: clinical indications and techniques. Equine Vet Edu. (2022) 34:432–424. 10.1111/eve.13489

[9] Tallon R McMillan M Ho N Dunkel B. Presumed generalised seizure following caudal epidural administration of morphine and detomidine in a pony. Equine Vet Educ. (2021) 33:e372–e5. 10.1111/eve.13342

[10] Gómez de Segura IA De Rossi R Santos M López San-Roman J Tendillo FJ San-Roman F. Epidural injection of ketamine for perineal analgesia in the horse. Vet Surg VS. (1998) 27:384–91. 10.1111/j.1532-950X.1998.tb00145.x 9662784

[11] Louro LF Milner PI Bardell D. Epidural administration of opioid analgesics improves quality of recovery in horses anaesthetized for treatment of hindlimb synovial sepsis. Equine Vet J. (2021) 53:682–9. 10.1111/evj.13338 32852063

[12] Natalini CC Robinson EP. Evaluation of the analgesic effects of epidurally administered morphine, alfentanil, butorphanol, tramadol, and U50488H in horses. Am J Vet Res. (2000) 61:1579–86. 10.2460/ajvr.2000.61.1579 11131602

[13] Valverde A Little CB Dyson DH Motter CH. Use of epidural morphine to relieve pain in a horse. The Canadian veterinary journal = La revue veterinaire canadienne. (1990) 31:211–2. 17423538 PMC1480787

[14] Skarda RT Muir WW III. Analgesic, hemodynamic, and respiratory effects induced by caudal epidural administration of meperidine hydrochloride in mares. Am J Vet Res. (2001) 62:1001–7. 10.2460/ajvr.2001.62.1001 11453471

[15] Natalini CC. Spinal anesthetics and analgesics in the horse. The veterinary clinics of North America. Equine Pract. (2010) 26:551–64. 10.1016/j.cveq.2010.07.005 21056299

[16] Leblanc PH Eberhart SW. Cardiopulmonary effects of epidurally administered xylazine in the horse. Equine Vet J. (1990) 22:389–91. 10.1111/j.2042-3306.1990.tb04301.x 2125264

[17] Valverde A. Alpha-2 agonists as pain therapy in horses. The veterinary clinics of North America. Equine Pract. (2010) 26:515–32. 10.1016/j.cveq.2010.07.003 21056297

[18] England GC Clarke KW. Alpha 2 adrenoceptor agonists in the horse–a review. Br Vet J. (1996) 152:641–57. 10.1016/S0007-1935(96)80118-7 8979422

[19] Aithal HP Amarpal Kinjavdekar P Pawde AM Pratap K. Analgesic and cardiopulmonary effects of intrathecally administered romifidine or romifidine and ketamine in goats (*Capra hircus*). J South Af Vet Assoc. (2001) 72:84–91. 10.4102/jsava.v72i2.623 11513266

[20] Amarpal Kinjavdekar P Aithal HP Pawde AM Pratap K. Analgesic, sedative and haemodynamic effects of spinally administered romifidine in female goats. J Vet Med A Physiol Pathol Clin Med. (2002) 49:3–8. 10.1046/j.1439-0442.2002.00385.x 11913823

[21] Fierheller EE Caulkett NA Bailey JV. A romifidine and morphine combination for epidural analgesia of the flank in cattle. Can Vet J La revue veterinaire canadienne. (2004) 45:917–23. 15600157 PMC545981

[22] Kinjavdekar P Aithal HP. Amarpal, Pawde AM, Pratap K, Singh GR. Potential effect of romifidine with lidocaine administration in goats. Small Rum Res. (2006) 64:293–304. 10.1016/j.smallrumres.2005.04.029

[23] Sarafzadeh RF Rezazadeh F Behfar M. Comparison of caudal epidural administration of lidocaine and xylazine to xylazine/ketamine combination in donkey (Equus asinus). Iran J Vet Surg. (2007) 2:7–16.

[24] Hamed MA Abouelnasr KS Ibrahim HM El-Khodery SA. Comparative, sedative, and analgesic effects of epidural dexmedetomidine and xylazine in donkeys (*Equus asinus*). J Equine Vet Sci. (2017) 59:104–9. 10.1016/j.jevs.2017.09.001 31952632

[25] Marzok MA El-khodery SA. Comparative analgesic and sedative effects of tramadol, tramadol-lidocaine and lidocaine for caudal epidural analgesia in donkeys (*Equus asinus*). Vet Anaesth Anal. (2015) 42:215–9. 10.1111/vaa.12195 24984816

[26] Elkammar M Gad S. Evaluation of the sedative, analgesic, clinicophysiological and haematological effects of intravenous detomidine, detomidine-butorphanol, romifidine, and romifidine-butorphanol in standing donkeys. Equine Vet Edu. (2014) 26:56. 10.1111/eve.12056

[27] El-Maghraby H Al-Akraa A Ghanem M. The sedative, analgesic and biochemical effects of romifidine in donkeys. Benha Vet Med J. (2005) 16:232–46.24688132

[28] Lizarraga I Janovyak E. Comparison of the mechanical hypoalgesic effects of five α2-adrenoceptor agonists in donkeys. Vet Rec. (2013) 173:294. 10.1136/vr.101684 23878192

[29] Lizarraga I Castillo-Alcala F Robinson LS. Sedative and mechanical antinociceptive effects of four dosages of romifidine administered intravenously to donkeys. Res Vet Sci. (2017) 112:46–51. 10.1016/j.rvsc.2017.01.010 28126600

[30] Marzok M Almubarak AI Abdel-Raheem SM El-Khodery S Shawaf T Kandeel M. Comparative study of the sedative and anti-nociceptive effects of sacrococcygeal epidural administration of romifidine, lidocaine, and romifidine/lidocaine in the dromedary camel. Front Vet Sci. (2022) 9:891581. 10.3389/fvets.2022.891581 35832332 PMC9271924

[31] Marzok M El-khodery S. Dose-dependent anti-nociceptive and sedative effects of epidural romifidine in cattle. Vet Record. (2016) 178:140. 10.1136/vr.103168 26787288

[32] Kariman editor. Cardiorespiratory and Analgesic Effects of Epidurally Administered Romifidine in the Horse Proceedings of 7th World Congress Vet Anaes. Berne: University of Berne (2000).

[33] Marzok M El-khodery SA. Comparative antinociceptive and sedative effects of epidural romifidine and detomidine in buffalo (*Bubalus bubalis*). Veterinary Record. (2017) 181:20. 10.1136/vr.103911 28386033

[34] DeRossi R Junqueira A Beretta M. Analgesic and systemic effects of xylazine, lidocaine and their combination after subarachnoid administration in goats. J S Afr Vet Assoc. (2005) 76:79–84. 10.4102/jsava.v76i2.402 16108526

[35] Abdel-Wahed R Abo-Ghanema I Nouh S Kenawy A Kassem M. Sedative, analgesic and clinico-biochemical effects of romifidine in donkeys. Kafrelsheikh Vet Med J. (2008) 6:132–45. 10.21608/kvmj.2008.115903

[36] Alkattan L Layth M Alkattan L. Analgesia and anesthesia with epidural xylazine/ ketamine in donkeys. Diag Therap Study. (2012) 1:37–44.

[37] Sinclair MD. A review of the physiological effects of alpha2-agonists related to the clinical use of medetomidine in small animal practice. Can Vet J La revue veterinaire canadienne. (2003) 44:885–97. 14664351 PMC385445

[38] Giovannitti JA Jr Thoms SM Crawford JJ. Alpha-2 adrenergic receptor agonists: a review of current clinical applications. Anesth Prog. (2015) 62:31–8. 10.2344/0003-3006-62.1.31 25849473 PMC4389556

[39] Cao D Heard K Foran M Koyfman A. Intravenous lipid emulsion in the emergency department: a systematic review of recent literature. J Emerg Med. (2015) 48:387–97. 10.1016/j.jemermed.2014.10.009 25534900

[40] Skarda R Muir W 3rd. Comparison of antinociceptive, cardiovascular, and respiratory effects, head ptosis, and position of pelvic limbs in mares after caudal epidural administration of xylazine and detomidine hydrochloride solution. Am J Vet Res. (1996) 57:1338–45. 8874730

[41] Aithal H Pratap AK Singh G. Clinical effects of epidurally administered ketamine and xylazine in goats. Small Ruminant Res. (1997) 24:55–64. 10.1016/S0921-4488(96)00919-417216318

[42] Butterworth JF Strichartz GR. The α2-adrenergic agonists clonidine and guanfacine produce tonic and phasic block of conduction in rat sciatic nerve fibers. Anaesth Anal. (1993) 76:295–301. 8093828

[43] Fikes LW Lin HC Thurmon JC. A preliminary comparison of lidocaine and xylazine as epidural analgesics in ponies. Vet Surg VS. (1989) 18:85–6. 10.1111/j.1532-950X.1989.tb01046.x 2929142

[44] Yaksh TL. Pharmacology of spinal adrenergic systems which modulate spinal nociceptive processing. Pharmacol Biochem Behav. (1985) 22:845–58. 10.1213/00000539-199205000-00016 2861606

[45] Sharma AK Kumar N Dimri U Hoque M Maiti SK Gupta OP. Romifidine-ketamine anaesthesia in atropine and triflupromazine pre-medicated buffalo calves. J Vet Med A Physiol Pathol Clin Med. (2004) 51:420–4. 10.1111/j.1439-0442.2004.00666.x 15610485

[46] Smith BD Baudendistel LJ Gibbons JJ Schweiss JF. A comparison of two epidural alpha 2-agonists, guanfacine and clonidine, in regard to duration of antinociception, and ventilatory and hemodynamic effects in goats. Anaesth Anal. (1992) 74:712–8. 1567040

[47] Murrell JC Hellebrekers LJ. Medetomidine and dexmedetomidine: a review of cardiovascular effects and antinociceptive properties in the dog. Vet Anaesth Anal. (2005) 32:117–27. 10.1111/j.1467-2995.2005.00233.x 15877658

[48] Kariman A Nowrouzian I Bakhtiari J. Caudal epidural injection of a combination of ketamine and xylazine for perineal analgesia in horses. Vet Anaesth Anal. (2000) 27115. 10.1046/j.1467-2995.2000.00018-4.x 28404052

